# Imaging Biomarkers To Predict Progression of Intermediate AMD with Avascular Pigment Epithelial Detachment in the University of Colorado AMD Registry

**DOI:** 10.21203/rs.3.rs-9193520/v1

**Published:** 2026-04-13

**Authors:** Soufiane Azargui, Andres Lisker-Cervantes, Jennifer L. Patnaik, Ramya Gnanaraj, Nihaal Mehta, Bill Gange, Anne M. Lynch, Alan G. Palestine, Marc T. Mathias, Niranjan Manoharan, Naresh Mandava, Talisa E. Carlo Forest

**Affiliations:** University of Colorado School of Medicine; University of Colorado School of Medicine; University of Colorado School of Medicine; University of Colorado School of Medicine; University of Colorado School of Medicine; University of Colorado School of Medicine; University of Colorado School of Medicine; University of Colorado School of Medicine; University of Colorado School of Medicine; University of Colorado School of Medicine; University of Colorado School of Medicine; University of Colorado School of Medicine

**Keywords:** avascular pigment epithelial detachment, intermediate age-related macular degeneration, advanced age-related macular degeneration, intraretinal hyperreflective foci, pigmentary changes, acquired vitelliform lesion, incomplete retinal pigment epithelium and outer retina atrophy

## Abstract

**Purpose:**

To investigate whether specific imaging biomarkers predict progression to neovascular AMD (nAMD) or geographic atrophy (GA) in eyes with high-risk intermediate age-related macular degeneration (iAMD) and avascular pigment epithelial detachment (PED).

**Methods:**

Prospective longitudinal cohort study of eyes with iAMD and avascular PED from the University of Colorado AMD registry (August 2014 - August 2023) with ≥1 month of follow-up through February 2024. Multimodal imaging, including color fundus photos (CFP), fundus autofluorescence (FAF), and optical coherence tomography (OCT), was graded by two reviewers for presence of specific imaging biomarkers. Time-to-progression survival analysis was conducted with hazard ratios calculated.

**Results:**

Over a median follow-up period of 35 months, 224 eyes (142 patients) were included. 31 (13.8%) eyes progressed to nAMD, and 63 (28.1%) to GA. Progression to nAMD was significantly predicted by pigmentary changes on CFP (HR=4.43 (95%CI: 1.77, 11.1), p=0.001) and intraretinal hyperreflective foci (iHRF) on OCT (HR=4.90 (95%CI: 2.02, 11.9), p=0.0005). Progression to GA was significantly predicted by pigmentary changes on CFP (HR=3.60 (95%CI: 1.94, 6.67), p<0.0001), iHRF (HR=4.13 (95%CI: 2.14, 7.94), p<0.0001), acquired vitelliform lesions (AVL; (HR=2.97 (95%CI: 1.55, 5.68), p=0.001)) and incomplete retinal pigment epithelium and outer retina atrophy (iRORA; (HR=4.18 (95%CI: 1.52, 11.4), p=0.006)). No other biomarkers demonstrated significance.

**Conclusion:**

In eyes with avascular PED, pigmentary changes and iHRF were significantly associated with progression to nAMD and GA, while AVL and iRORA were specifically to GA. We highlight in this study important imaging biomarkers that help identify high-risk eyes that may warrant closer monitoring to ensure timely therapeutic intervention.

## Introduction

Age-related macular degeneration (AMD) is the leading cause of irreversible vision among the elderly population in industrialized nations. Its prevalence is expected to increase to 288 million by 2040.^[1, 2]^ Retinal pigment epithelial detachments (PED), occurring between the basal lamina of the retinal pigment epithelial (RPE) cell and the inner collagenous Bruch’s membrane, commonly accompany AMD.^[3–6]^ Avascular PEDs represent a distinct subtype arising from the gradual enlargement and coalescence of pre-existing soft drusen.^[7, 8]^ These PEDs are clinically important because they are known to increase the risk of progression to the late forms of AMD: neovascular AMD (nAMD) and geographic atrophy (GA).^[9–12]^ Despite the well-known risk, it remains challenging to predict how quickly a given avascular PED will progress and, critically, to which late AMD form it will progress. Current prognostic tools do not reliably discriminate risk paths between these two forms or provide meaningful temporal information. This has growing clinical relevance given the emergence of novel therapies, such as the Valeda Light Delivery System photobiomodulation therapy, which have made it more important to identify which intermediate AMD (iAMD) patients are at the highest risk for progression and may benefit most from early intervention.^[13–17]^

Although prior studies have examined progression from iAMD to advanced AMD, iAMD encompasses a broad spectrum, and later phenotypes such as those with avascular PEDs have received limited focused investigation, despite their higher risk of progression and the potential value of targeted prediction models.^[5, 18–21]^ In this study, we investigate whether specific imaging biomarkers can predict faster progression of iAMD eyes with avascular PED to nAMD or GA.

## Methods

This prospective longitudinal study was approved by the Colorado Multiple Institutional Review Board and follows the ethical principles of the Declaration of Helsinki. The study includes patients from the University of Colorado AMD registry, developed by the Department of Ophthalmology at the University of Colorado School of Medicine. Detailed descriptions of the recruitment process, consent procedures, and inclusion and exclusion criteria for patients included in the AMD registry are available in prior publications.^[18, 22–24]^ Recruitment into this registry started on July 9, 2014 and recruitment is ongoing. Inclusion criteria include participants aged 50–99 years with AMD in one or both eyes and the ability to provide informed consent. The exclusion criteria include a history of concomitant retinal and uveitic disease.^[23]^

### Multi-Modal Imaging

Each patient is consented to the collection of medical and ocular history, and review of multimodal imaging (MMI) data collected, including color fundus photos (CFP), fundus autofluorescence (FAF), and optical coherence tomography (OCT). OCT angiography and fluorescein angiography (FA) are also obtained at the discretion of the retina provider. The MMI protocol is as follows: the Eidon True Color Confocal Scanner (iCare Spa, Vantaa, Finland) or Topcon TRC-50DX (Topcon, Tokyo, Japan) device is used to obtain a 60 degree CP centered on the fovea (field 2) and the Heidelberg Spectralis (Heidelberg Engineering Inc) device was used to obtain a 30 or 60 degree FAF with automatic real time-function (ART) of 50 centered on the fovea. The Heidelberg Spectralis device is also used to obtain high resolution horizontal and vertical enhanced-depth imaging line scans (6 mm/30 degree/ART 100) and a 49-line cube scan (6 mm/30 degree/ART 9) centered on the fovea.^[18, 25]^

### The iAMD longitudinal cohort

For this specific study, we limited our analysis to patients with iAMD at enrollment (July 2014 to August 2023) with avascular PED and at least 1 month of follow-up through February 2024. Two retina specialists confirmed the diagnosis of iAMD at enrollment in at least one eye of a patient (and no advanced AMD in either eye), using the classification described by the Beckman Initiative for Macular Research Committee.^[26]^ Two trained graders reviewed the MMI for presence of avascular PED in each eye. The medical records and MMI of each iAMD eye with avascular PED were then reviewed for date of progression to advanced AMD (defined by the presence of GA or choroidal neovascularization per the Beckman classification), or date of last retina clinic visit if no evidence of progression.

### Qualitative Imaging Biomarkers

The MMIs of patients in the cohort were reviewed by two trained graders for baseline qualitative imaging biomarkers, with any discrepancies resolved by a third trained grader. CFP was evaluated for the presence or absence of hard drusen, soft drusen, calcified drusen, cuticular drusen, pigmentary changes and reticular pseudodrusen (RPD). FAF images were specifically graded for the presence or absence of RPD. OCT images were assessed for the presence or absence of outer retinal tubulations (ORT), retinal pseudocysts, non-neovascular subretinal fluid (nnSRF), acquired vitelliform lesion (AVL), RPD, intraretinal hyper-reflective foci (iHRF) and incomplete retinal pigment epithelium and outer retina atrophy (iRORA).

### Statistical analysis

Statistical analysis was performed using SAS version 9.4 (Cary, North Carolina) software. Characteristics of the cohort, including patient demographics and clinical variables, were compiled with basic frequencies and percentages, along with mean and standard deviations (SD) calculated for continuous variables which were normally distributed and medians and ranges for follow-up time. A survival analysis with time to progression specific to each eye was conducted with hazard ratios calculated. Cox proportional hazard modeling with competing risks was utilized to determine hazard ratios for time to conversion for each type of advanced AMD. Sandwich estimators were included to account for the correlation of the two eyes being included. The p-value cut-point was set at < 0.05.

## Results

Two hundred twenty-four eyes with avascular PEDs of 142 patients were included in this study. The median follow-up period was 35.2 months (range 1.4–118.6). The mean age of patients at study enrollment was 75.5 years (SD = 6.9). There were 47 (33%) male patients and a high proportion of Caucasian patients (135/142, 95%). Average BMI was 27.5 (SD = 5.7). Eighty-five patients (60%) had no history of smoking, 3 (2%) were current smokers, and 54 (38%) were former smokers. Fifty-nine patients (42%) have a known family history of AMD.

Thirty-one (13.8%) eyes progressed to nAMD, while 63 (28.1%) eyes progressed to GA. In our cohort, soft drusen, hard drusen, and iHRF were the most common imaging findings (Table 1), with similar prevalences among eyes that progressed to nAMD and those that progressed to GA. There were no statistically significant differences in the prevalences of imaging biomarkers between eyes that converted to nAMD and those that progressed to GA.

Among iAMD eyes with avascular PED, time to progression to nAMD was significantly predicted by the presence of pigmentary changes on CFP (HR = 3.39 (95% CI: 1.47, 7.84), p = 0.004) and iHRF on OCT (HR = 3.89 (95% CI: 1.72, 8.80), p = 0.001). Progression to GA was also significantly predicted by presence of pigmentary changes on CFP (HR = 3.78 (95% CI: 2.08, 6.85), p < 0.0001) and iHRF on OCT (HR = 4.14 (95% CI: 2.25, 7.62), p < 0.0001), in addition to AVL (HR = 3.07 (95% CI: 1.67, 5.66), p = 0.0003) and iRORA (HR = 3.57 (95% CI: 1.45, 8.80), p = 0.006) on OCT. No other imaging biomarkers demonstrated a statistically significant association with progression. (Table 2)

## Discussion

In this iAMD cohort specifically with avascular PEDs, several MMI biomarkers were significantly associated with progression to advanced AMD. Prior AREDS analyses have shown that avascular PEDs confer substantial risk for late AMD, with 42% of eyes progressing within five years (19% to GA and 23% to nAMD), underscoring avascular PED as a high-risk phenotype with heterogeneous progression pathways.^[20, 27–29]^ The natural history of avascular PEDs generally follows one of three trajectories—collapse with subsequent atrophy, vascularization with exudation, or long-term persistence. In our cohort, pigmentary abnormalities on color fundus photography and iHRF on OCT were associated with progression to both GA and advanced neovascular disease, whereas AVL and iRORA were more specifically associated with progression along the non-neovascular atrophic pathway.

Prior studies have shown that large drusen (≥ 125 μm) in the central macula, especially when accompanied by pigmentary abnormalities on color fundus photography, significantly increase the risk of progression to advanced AMD. Eyes with RPE hyperpigmentation and large drusen have a higher 5-year risk of developing either nAMD or GA, while eyes with RPE depigmentation are particularly prone to progressing to GA.^[30]^ Similarly, iHRF is a key OCT biomarker of AMD progression, defined as discrete, well-demarcated hyperreflective lesions within the neurosensory retina, measuring ≥ 3 pixels and with reflectivity equal to or greater than the RPE band.^[28, 31]^ Overlying iHRF is frequently observed with avascular PED, often preceding overt structural breakdown by more than a year and correlating with increased risk of progression.^[11, 19]^ Verma et al. reported a 21% higher odds of progression to advanced AMD over 24 months in iAMD eyes with iHRF, while Wakatsuki et al. reported more than a twofold higher odds of progression to nAMD.^[28, 31]^ Although these studies did not specifically isolate eyes with avascular PEDs, they collectively support iHRF as a marker of increased progression risk. Notably, in our avascular PED subset, the presence of iHRF conferred substantially higher risk, with over a fourfold increase in odds of progression to GA and a nearly fourfold increase in odds of progression to nAMD, exceeding effect sizes previously reported in broader iAMD cohorts. The higher risk observed in this subgroup likely reflects phenotypic enrichment within the avascular PED cohort, in which the coexistence of iHRF indicates greater structural instability, RPE dysfunction, and heightened disease activity. Our finding that pigmentary change and iHRF were statistically significant predictors of progression to both GA and nAMD is concordant with prior observations and reinforces the prognostic relevance of these biomarkers in risk stratification.

In regard to discriminating between which advanced form of AMD an avascular PED will progress to, our study elucidated imaging biomarkers for progression specifically to GA but none exclusively to nAMD. Prior work has examined the prognostic value of these biomarkers for GA progression, AVL and iRORA. AVLs are considered markers of active RPE dysfunction and impaired metabolic exchange between the RPE and overlying neurosensory retina.^[18, 32]^ Our finding that AVL significantly predicts progression to GA in iAMD eyes with avascular PEDs is consistent with prior reports in more heterogenous iAMD cohorts.^[25]^ Mahmoudi et al. observed that approximately 10% of iAMD eyes with AVL progressed to atrophy within 24 months, while earlier studies reported a broader 15–50% risk of AVL collapse to cRORA.^[33]^ These prior studies did not specifically isolate eyes with avascular PEDs. Therefore, our results build upon prior work, indicating that AVL confers elevated progression risk in the setting of avascular PED.

Existing evidence has established iRORA as an atrophic precursor lesion on OCT that frequently progresses to cRORA, a defining feature of GA^[18, 34–36]^ Reported progression rates have been high, with Corradetti et al. showing that over 90% of eyes with incident iRORA progressed to cRORA within 24 months, and Wu et al. reporting a markedly increased risk of GA in eyes with prevalent or incident iRORA (adjusted HR ≈ 12).^[34, 36]^ In contrast, within our avascular PED subset, progression risk appeared substantially lower in the present study: only 14% (9/63) of eyes with both avascular PED and iRORA progressed to GA (HR = 3.57, p = 0.006) during the study period (approximately 3 years). The difference in progression rates between these studies could be due to different patient populations, study follow-up variability, or different thresholds for iRORA classification. Alternatively, iRORA occurring in the presence of an avascular PED may represent an earlier stage of atrophy formation due to persistence of the PED, with a distinct risk profile and potentially slower atrophic trajectory than that reported in broader iAMD cohorts.

Strengths of this study include its prospective longitudinal design, the use of well-established enrollment criteria from the University of Colorado AMD Registry, and a standardized multimodal image review process. Trained graders performed AMD staging and image biomarker assessment, with a third independent grader used for adjudication to support consistency and accuracy. In addition, our analysis focused on a specific, well-defined subset of high-risk iAMD eyes, offering a novel perspective within a population that has otherwise not been broadly studied.

Several limitations should be acknowledged. This was a single academic center study, and although the overall cohort was large, the number of eyes progressing to advanced AMD was relatively small, with a median follow-up of approximately 3 years. This may have limited our study’s power to uncover significant biomarkers, particularly for progression to nAMD since the sample size was smaller. Furthermore, fluorescein angiography and OCT angiography were not obtained in all patients, as their use was at the clinician’s discretion. These modalities were employed in equivocal cases and during research review for progression assessment, and longitudinal multimodal imaging was carefully evaluated to monitor for interval fluid changes suggestive of neovascular conversion. Despite these safeguards, a small number of fibrovascular PEDs without exudation could have been misclassified as avascular PEDs and inadvertently included in the analysis. Finally, while the study was conducted longitudinally, the imaging biomarkers were evaluated only cross-sectionally at enrollment. A longitudinal evaluation of how these biomarkers evolve during the disease progression would provide further information on AMD pathogenesis. Additionally, incorporating quantitative assessment such as PED size metrics or drusen volume may further refine risk stratification. Finally, future studies should explore the use of artificial intelligence to enhance predictive modeling of AMD progression in this high risk iAMD population, potentially incorporating multimodal imaging biomarkers, genetics, and electronic health record data.

In conclusion, among eyes with iAMD and avascular PEDs, pigmentary abnormalities and iHRF were strongly associated with progression to both GA and nAMD, while AVL and iRORA were more specifically linked to progression to GA. These findings refine risk stratification within a high-risk phenotype and highlight the value of multimodal imaging biomarkers in anticipating disease trajectory and informing surveillance strategies.

## Figures and Tables

**Table 1:** Comparison of prevalence of imaging biomarkers between eyes with avascular PED that progress to nAMD or GA

Qualitative Imaging Biomarkers	Total number of eyes with the imaging biomarker (%) N = 224	Progression to advanced nAMD n = 31	Progression to GA n = 63	
		Eyes with the imaging biomarker (%)	Eyes with the imaging biomarker (%)	p-value
CFP Hard Drusen	179/213 (84.0%)	26/31 (83.9%)	55/63 (90.2%)	0.39
CFP Soft Drusen	204/213 (95.8%)	30/31 (96.8%)	60/63 (98.4%)	0.63
CFP RPD	23/213 (10.8%)	2/31 (6.4%)	6/63 (9.8%)	0.60
CFP Calcified Drusen	17/213 (8.0%)	1/31 (3.2%)	4/63 (6.6%)	0.51
CFP Cuticular Drusen	12/213 (5.6%)	1/31 (3.2%)	0/63 (0%)	NC
CFP Pigmentary Change	117/213 (54.9%)	21/31 (67.7%)	42/63 (68.8%)	0.92
FAF RPD	34/216 (15.7%)	5/31 (16.1%)	11/63 (17.5%)	0.86
OCT ORT	1/224 (0.4%)	0/31 (0%)	1/63 (1.8%)	NC
OCT Retinal Pseudocysts	2/223 (0.9%)	0/31 (0%)	2/63 (3.2%)	NC
OCT nnSRF	4/224 (1.8%)	1/31 (3.2%)	2/63 (3.2%)	0.99
OCT AVL	34/224 (15.2%)	6/31 (19.4%)	15/63 (23.8%)	0.58
OCT RPD	29/224 (13.0%)	5/31 (16.1%)	11/63 (17.5%)	0.86
OCT iHRF	130/224 (58.0%)	22/31 (71.0%)	44/63 (69.8%)	0.92
OCT iRORA	22/224 (9.8%)	3/31 (9.7%)	9/63 (14.3%)	0.55

NC = not calculable due to zero cell size

CFP - Color Fundus Photos

FAF - Fundus Autofluorescence

OCT - Optical Coherence Tomography

RPD - Reticular Pseudodrusen

ORT - Outer Retinal Tubulation

nnSRF - Non-neovascular Subretinal Fluid

AVL - Acquired Vitelliform Lesion

iHRF - Intraretinal Hyperreflective Foci

iRORA – Incomplete Retinal Pigment Epithelial and Outer Retinal Atrophy

**Table 2:** The relative hazard of progression for presence of imaging biomarkers to either of the advanced forms of age-related macular degeneration in eyes with avascular PED

Qualitative Imaging Biomarkers		Total number of eyes with the imaging biomarker (%)	Progression to nAMD		Progression to GA	
			HR (95% CI)	p-value	HR (95% CI)	p-value
CFP Hard Drusen		179/213 (84.0%)	0.67 (0.23, 1.96)	0.469	1.19 (0.44, 3.20)	0.731
CFP Soft Drusen		204/213 (95.8%)	1.25 (0.14, 10.9)	0.840	2.61 (0.34, 19.9)	0.355
CFP RPD		23/213 (10.8%)	0.64 (0.20, 2.02)	0.448	1.17 (0.39, 3.52)	0.779
CFP Calcified Drusen		17/213 (8.0%)	0.78 (0.10, 6.09)	0.813	1.83 (0.50, 6.71)	0.363
CFP Cuticular Drusen		12/213 (5.6%)	0.92 (0.10, 8.16)	0.940	NC	-
CFP Pigmentary Change		117/213 (54.9%)	3.39 (1.47, 7.84)	**0.004**	3.78 (2.08, 6.85)	**< 0.0001**
FAF RPD		34/216 (15.7%)	1.23 (0.52, 2.93)	0.636	1.60 (0.75, 3.42)	0.225
OCT ORT		1/224 (0.4%)	NC	-	NC	-
OCT Retinal Pseudocysts		2/223 (0.9%)	NC	-	NC	-
OCT nnSRF		4/224 (1.8%)	3.10 (0.31, 30.9)	0.336	3.04 (0.43, 21.5)	0.266
OCT AVL		34/224 (15.2%)	2.02 (0.86, 4.78)	0.108	3.07 (1.67, 5.66)	**0.0003**
OCT RPD		29/224 (13.0%)	1.43 (0.60, 3.38)	0.421	1.92 (0.89, 4.16)	0.097
OCT iHRF		130/224 (58.0%)	3.89 (1.72, 8.80)	**0.001**	4.14 (2.25, 7.62)	**< 0.0001**
OCT iRORA	22/224 (9.8%)		1.95 (0.54, 7.11)	0.312	3.57 (1.45, 8.80)	**0.006**

HR – hazard ratio

CI – confidence interval

NC – not calculable due to zero cell size

CFP – Color Fundus Photos

FAF – Fundus Autofluorescence

OCT – Optical Coherence Tomography

RPD – Reticular Pseudodrusen

ORT – Outer Retinal Tubulation

nnSRF – Non-neovascular Subretinal Fluid

AVL – Acquired Vitelliform Lesion

iHRF – Intraretinal Hyperreflective Foci

iRORA – Incomplete Retinal Pigment Epithelial and Outer Retinal Atrophy

## Key Message

### What is known

Avascular PEDs confer substantial risk for late forms of AMD. But these studies of intermediate AMD eyes have not specifically isolated those with avascular PEDs to better understand this high risk subset.

This study highlights important imaging biomarkers, such as acquired vitelliform lesions, that can help predict which eyes with avascular PEDs may progress to the advanced vision-threatening forms of AMD and may therefore warrant closer monitoring to ensure timely therapeutic intervention.

These findings refine risk stratification within this high-risk phenotype and highlight the value of multimodal imaging biomarkers in anticipating disease trajectory and informing surveillance strategies
